# Fertility Preservation and Restoration Options for Pre-Pubertal Male Cancer Patients: Current Approaches

**DOI:** 10.3389/fendo.2022.877537

**Published:** 2022-06-16

**Authors:** Elena Eugeni, Iva Arato, Rachele Del Sordo, Angelo Sidoni, Andrea Garolla, Alberto Ferlin, Riccardo Calafiore, Stefano Brancorsini, Francesca Mancuso, Giovanni Luca

**Affiliations:** ^1^ Department of Medicine and Surgery, University of Perugia, Perugia, Italy; ^2^ Department of Medicine and Medical Specialties, Division of Medical Andrology and Endocrinology of Reproduction, University of Terni, Terni, Italy; ^3^ Division of Anatomic Pathology and Histology, Department of Experimental Medicine, University of Perugia, Perugia, Italy; ^4^ Unit of Andrology and Reproductive Medicine, Department of Medicine, School of Medicine and Surgery, University of Padua, Padua, Italy; ^5^ Section of Pathology (Terni), Department of Medicine and Surgery, University of Perugia, Perugia, Italy; ^6^ International Biotechnological Center for Endocrine, Metabolic and Embryo-Reproductive Translational Research (CIRTEMER), Department of Medicine and Surgery, University of Perugia, Perugia, Italy

**Keywords:** fertility, spermatogonial cell, gonadotoxic cancer treatment, cryopreservation, testicular tissue transplantation, SSCs transplantation, *de novo* morphogenesis, *In vitro* spermatogenesis

## Abstract

Fertility preservation for prepubertal male patients undergoing gonadotoxic therapies, potentially depleting spermatogonial cells, is an expanding necessity, yet most of the feasible options are still in the experimental phase. We present our experience and a summary of current and novel possibilities regarding the different strategies to protect or restore fertility in young male patients, before proceeding with chemotherapy or radiotherapy for malignances or other diseases. Adult oncological patients should always be counselled to cryopreserve the semen before starting treatment, however this approach is not suitable for prepubertal boys, who aren’t capable to produce sperm yet. Fortunately, since the survival rate of pediatric cancer patients has skyrocketed in the last decade and it’s over 84%, safeguarding their future fertility is becoming a major concern for reproductive medicine. Surgical and medical approaches to personalize treatment or protect the gonads could be a valid first step to take. Testicular tissue autologous grafting or xenografting, and spermatogonial stem cells (SSCs) transplantation, are the main experimental options available, but spermatogenesis *in vitro* is becoming an intriguing alternative. All of these methods feature both strong and weak prospects. There is also relevant controversy regarding the type of testicular material to preserve and the cryopreservation methods. Since transplanted cells are bound to survive based on SSCs number, many ways to enrich their population in cultures have been proposed, as well as different sites of injection inside the testis. Testicular tissue graft has been experimented on mice, rabbits, rhesus macaques and porcine, allowing the birth of live offspring after performing intracytoplasmic sperm injection (ICSI), however it has never been performed on human males yet. *In vitro* spermatogenesis remains a mirage, although many steps in the right direction have been performed. The manufacturing of 3D scaffolds and artificial spermatogenetic niche, providing support to stem cells in cultures, seems like the best way to further advance in this field.

## Introduction

The increasing incidence of cancer during childhood and the rising survival rate, currently estimated around 84% after 5 years from diagnosis (1), is leaving behind a large population of young male patients whose fertility is at stake (2, 3). The most common cancers in children are leukemias, lymphomas, tumors involving the brain or CNS, bone or soft tissue sarcomas, germ cell tumors, and embryonal tumors (4)

In adult patients, cryopreservation of seminal fluid is a safe and proven approach to preserve fertility prior to initiating gonadotropic treatments and should be routinely proposed by the caregiver in consultation with a reproductive medicine specialist (5, 6). Pre-pubertal patients are not capable yet of producing spermatozoa; therefore, this approach is not sustainable in their course of treatment (7, 8).

Although several valid studies have been published in recent years regarding methods to protect or restore fertility in children, and some practices are now likely to be ready for clinical use, these options still remain exclusive to the experimental field.

It is estimated that about half of adult patients with an history of pediatric malignancy will have difficulty conceiving children, with a major impact on their quality of life (9–11).

A variety of oncological treatments could threaten testicular function (12–16), such as surgery, chemotherapy, radiotherapy, or combination therapy, with potential synergistic effects in causing gonadal toxicity. In the pre-pubescent male patient, the seminiferous tubules are populated by Spermatogonial Stem Cells (SSCs) which, being actively proliferating, are particularly sensitive to damage by chemotherapy or radiotherapy (17, 18). A fraction of SSCs is not rapidly proliferating and constitutes a reserve of stem cells. Such cells, referred as A dark spermatogonia or State 0 SSCs, have been widely investigated over the years and are expressed in higher percentage in the testis of humans and non-human primates than in rodents (22% vs 0.3%). These SSCs are less chemosensitive, but their damage might lead to a condition of irreversible infertility, as the pool of SSCs is no longer able to proliferate and subsequently differentiate (19, 20).

Several chemotherapeutic agents have been associated with risk of testicular toxicity, mainly alkylating agents (19–21), platinum agents (22, 23) or cytarabine (21). Therapy with cyclophosphamide or the combination of chlormethine and procarbazine may cause alterations in spermatogenesis, and this risk increases as the dose increases (21). Other chemotherapies that may be implicated in spermatogenesis damage include ifosfamide, busulfan/cyclophosphamide or fludarabine/melphalan, used in some protocols for Hematopoietic stem-cell transplantation (HSCT) conditioning, although studies that evaluate the specific adverse effects for some of them are lacking and their toxicity is only deemed as probable (21). Risk assessment of the impact of chemotherapy on spermatogenesis is not straightforward, as many protocols involve the administration of several drugs together or in combination with radiotherapy, and the patient’s age and follow-up time are also relevant, given the potential recovery of spermatogenetic capacity after a period of time. Even taking all these elements into account, individual patient variability and genetic predisposition may play a major role in the gonadotoxic effect of therapy (21).

Leydig cells are more resistant to the toxic action of chemotherapeutic agents and their function is generally preserved (24). Combined treatments with alkylating agents and pelvic radiotherapy, however, may impair their function, bringing to a clinical condition characterized by increased LH and decreased Testosterone (25). Pre- hematopoietic cell transplantation conditioning protocols and treatments including chemotherapy and irradiation are generally capable of damaging both germ cells and Leydig cells (26, 27).

Given their known toxicity, alkylating agents are used with caution in pediatric oncology protocols, either by attempting to reduce the cumulative dose or by choosing drugs with a more favorable harmful profile (28), but this is often not feasible in cancer in advanced stages. The risk of testicular toxicity increases when multiple alkylating agents are used together, when treatments are prolonged, or when the patient is young (28).

Several scores such as alkylating agent dose (AAD) (29) or cyclophosphamide equivalent dose (CED) (30) are available to quantify exposure to alkylating agents and assess the risk of potential adverse events, but they do not account for all drugs currently in use. The recovery of spermatogenesis after therapy depends on the ability of quiescent SCCs to survive and resume differentiation, so the duration of azoospermia increases progressively depending on the extent of damage and the scarcity of the residual stem cell population (31).

Radiation therapy is also capable of damaging the delicate SCCs, as the germinal epithelium is very sensitive to radiation. Cranial radiotherapy could also damage the hypothalamic-pituitary region and cause a form of central hypogonadism, triggered by impaired stimulation of the testis by the lack of LH and FSH. Even doses of 0.1 Gy can temporarily alter spermatogenesis (32, 33), while doses greater than 6 Gy permanently damage the subject’s spermatogenetic capacity causing irreversible azoospermia (34). Gonadotoxic protocols include abdomen, pelvis, and total body irradiation, total node irradiation, and cranial radiotherapy, which can cause alterations in the pituitary- testicle axis, leading to hypogonadotropic hypogonadism if administered at doses above 35-40 Gy (21). Leydig cells are more resistant to these effects, but even fractional doses of testicular irradiation of 12 Gy can increase LH values in pre-pubertal patients, thus suggesting a toxic effect (35). Doses greater than 20 Gy generally require hormone replacement therapy to achieve normal pubertal development. In the adult male, however, the irreversibly toxic dose is greater than 30 Gy (36).

The overall gonadotoxic effect of radiation therapy is related to total dose, irradiated volume, fractionated dose, and patient age (37).

Green et al. (38) demonstrated that patients exposed at pre-pubescent age to testicular radiotherapy at cumulative doses > 7.5 Gy, an AAD > 2, or treatment with procarbazine or high dose of cyclophosphamide showed reduction in their ability to procreate. Specifically, patients included in the study who survived childhood cancers were half as likely to produce offspring as their siblings (Hazard Ratio of pregnancy of 0.56 versus 0.91). Treatment doses and patterns are relevant to this risk, as is age at the diagnosis.

Unilateral orchiectomy for the treatment of testicular tumors can reduce the number of germ cells available, but it is not generally associated with azoospermia. An observational study showed that 85% of patients who underwent unilateral orchiectomy were able to procreate during the subsequent 11-year follow-up (13). The combination of surgical treatment, chemotherapy and radiation therapy increases the risk of long-term gonadotoxicity in the child.

Preserving and protecting the fertility of young cancer patients is now a shared goal within their treatment plan, but nevertheless many doubts still remain about which strategies should be proposed to the patient and family, as many approaches are still considered in the experimental and research phase.

**Figure 1:**
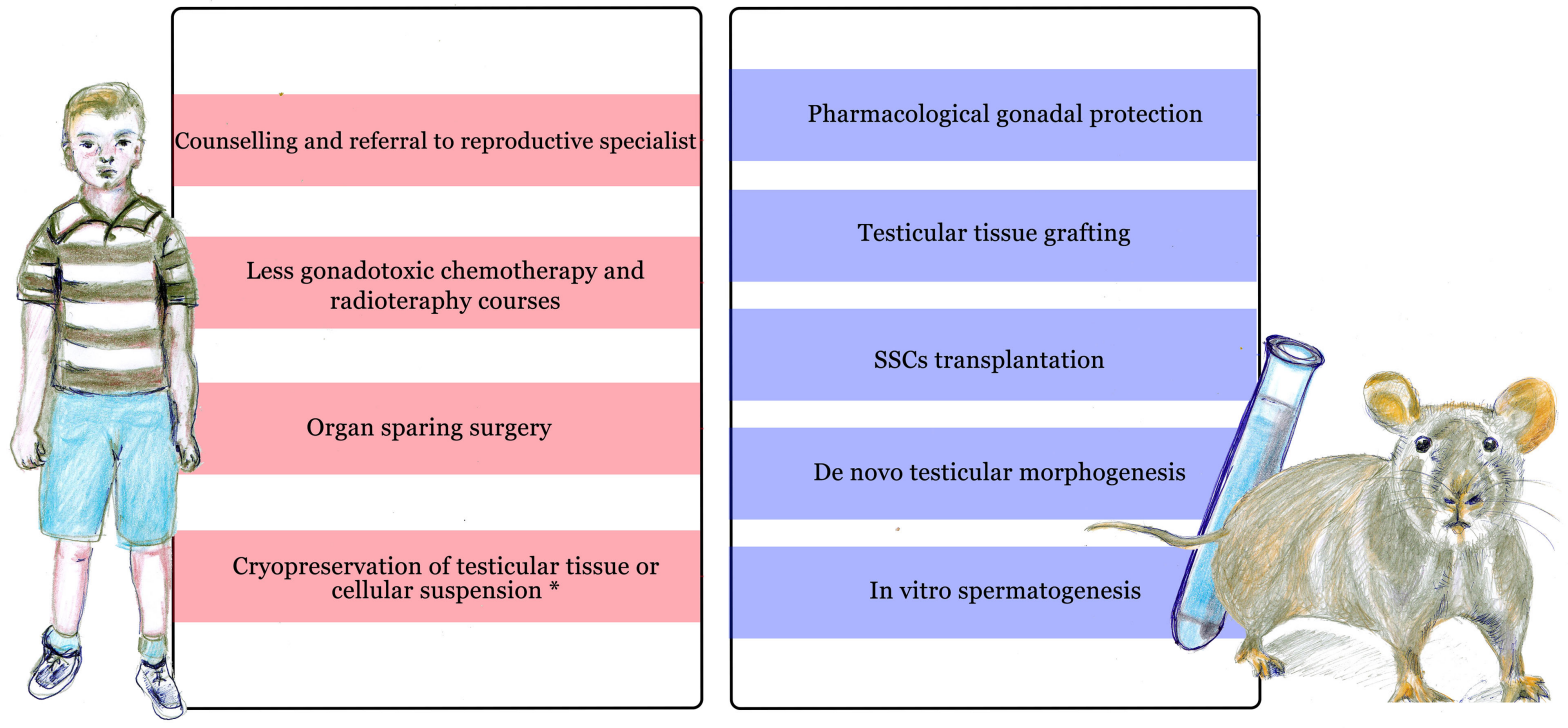
Current approaches to preserve and restore fertility in prepubertal males undergoing cancer treatment. On the left side: current clinical approaches available before and during treatment. On the right side: experimental methods, mainly tested on animal models (including rodents, non human primates and others) or *in vitro*. *Cryopreservation of testicular material is only available in selected centers during experimental protocols.

Current guidelines (5, 6) recommend informing the patient and family about the potential infertility risk of planned therapies and referring them to a reproductive medicine specialist at the earliest possible opportunity, to help them to evaluate the available options for preserving future fertility (**Figure 1**).

## Pharmacological Approaches to Preserve Testicular Function

One of the hypothesized gonadoprotective strategies is the use of molecules capable of inhibiting the pituitary secretion of LH and FSH, the hormones that stimulate the testis to produce testosterone and spermatozoa. Agonists or antagonists of the pituitary receptor of GnRH are able to block this hormonal production, generating a state of hypogonadotropic hypogonadism that could be exploited to protect the gonads. However, the use of GnRH agonist or antagonist for gonado-protective purposes during or before treatment for neoplasms does not appear to be useful in humans, and it is not recommended in ASCO guidelines. Such a strategy had appeared promising following some studies in rats (39–41) in which administration of GnRH before, during, or after therapy with alkylating agents or radiotherapy resulted in a marked increase in proliferating germ cells and a resumption of spermatogenic capacity. A similar effect has not been demonstrated in humans in several studies in which GnRH antagonist was associated with Testosterone (42–45). A single study (46) in which only Testosterone was administered showed positive results, although under conditions, as in the treatment of nephrotic syndrome and during therapy with cyclophosphamide alone (46). Several studies in nonhuman primates have confirmed this disappointing fact (47). However, GnRH agonist treatment seems to have a positive effect on the success of SCC transplantation, as proven in rats (48, 49). Testosterone suppression induced by such treatment, however, may induce an increased immune response (50), and this may justify the conflicting data obtained in the same pre-transplant treatment in nonhuman primates (47).

Some *in vivo* and *in vitro* studies in animal models have tested the protective effect of anti-apoptotic substances, such as sphingosine-1-phosphate (51) or immunomodulatory substances such as AS101 (52). In mice, these compounds offer some testicular protection against radiation or cyclophosphamide damage, but no relevant effect has been demonstrated in humans so far. Similar approaches have been used to test the protective effect of L- Carnitine (53), and several antioxidant substances, including curcumine nanocrystals (54), Moringa oleifera (55), alpha-tocopherol-succinate (56) and ascorbic acid (57), all tested on the gonads of cyclophosphamide-exposed rodents, with encouraging results but yet to be proven in humans.

The rationale behind the use of these substances is that some chemotherapy drugs, such as cyclophosphamide, are capable of generating radical oxygen species (ROS) and causing cellular apoptosis or altering DNA synthesis (51–56). Oxidative stress can activate enzymes such as sphingomyelinases, which can release ceramide from cell membranes and trigger cell apoptosis, and substances such as S1P might inhibit this specific process (51). A different detrimental effect of some chemotherapy drugs is the fragmentation of cellular DNA, resulting in an abnormal chromatin structure, a condition that reduces seminal quality and is known to decrease fertility (52). Immunomodulatory substances capable of limiting this alteration would be very useful for their gonadoprotective action. On the other hand, the administration of substances with antioxidant power may be able to reduce the oxidative stress produced by chemotherapic agents such as cyclophosphamide, which also seems to be able to damage the structure of the blood-testis barrier, altering the expression of Occludin proteins, produced by Sertoli cells (53).

**Figure 2:**
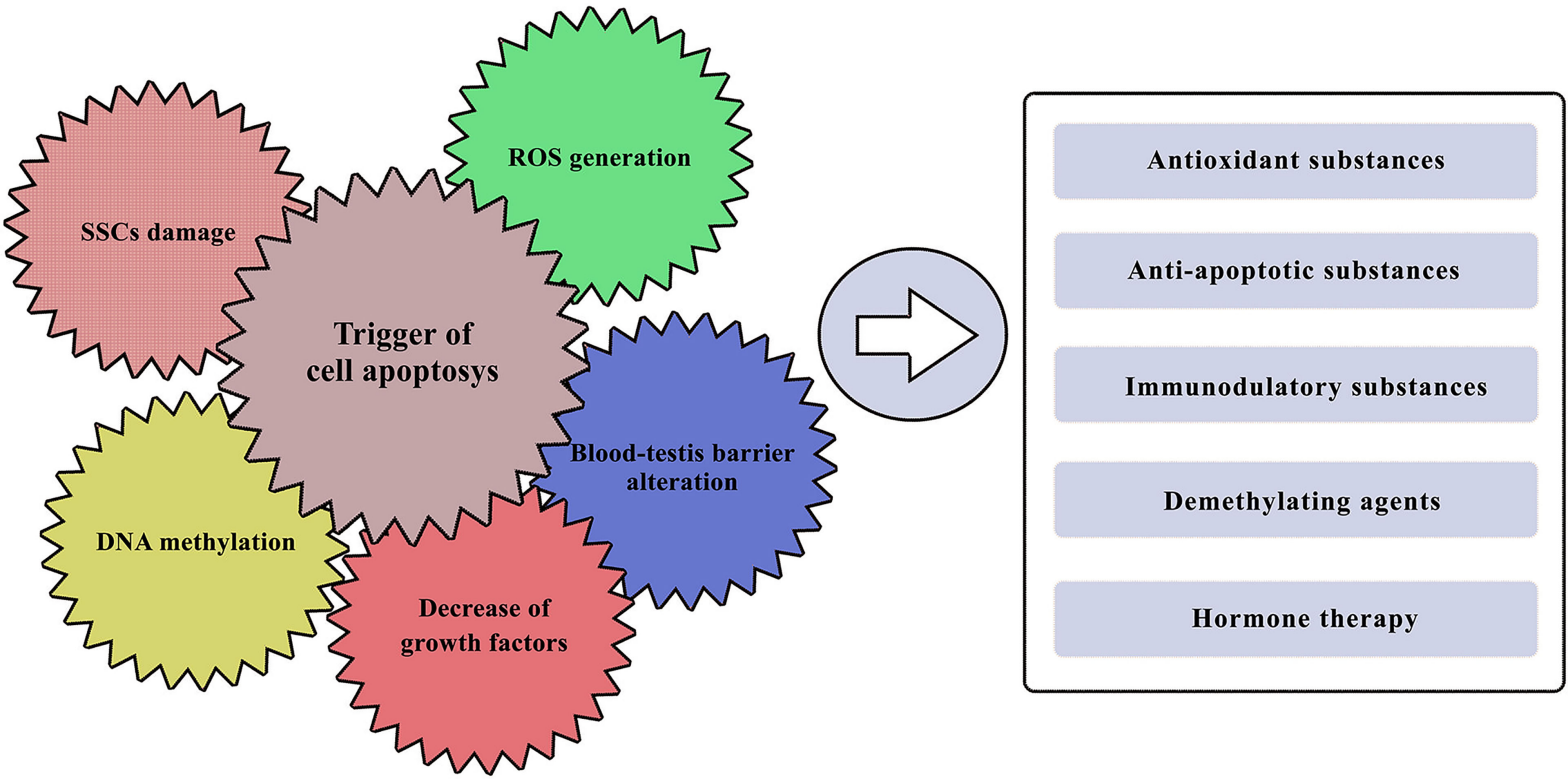
Known gonadotoxic damage of cancer treatments and potential pharmacological approaches. On the left side: the major effects with which various chemotherapeutic agents could impair testicular function. On the right side: potential pharmacological approaches that have been tested so far, with more or less promising results in protecting the male gonad.

To understand how to pharmacologically protect the testis in pre-pubertal children, it is thus essential to study the mechanisms involved in cytotoxic damage and survival of the SSCs population, as well as understanding the functioning of the complex spermatogenetic niche (**Figure 2**). The ability of germinal spermatogonial cells to ensure a continuous population of cells that can differentiate is essential for spermatogenic capacity (58). Several studies have investigated the recovery capabilities of spermatogonial stem cells after chemotherapeutic damage (59, 60). Many papers published by Parker et al. (61, 62) have focused on the effect of glial cell line- derived neurotrophic factor (GDNF) produced by Sertoli cells and essential for the survival of SSCs. GDNF is a member of the TGF-β superfamily, and by binding to its receptor and RET/GFRA1 on SCCs it regulates their survival and differentiation (63, 64).

Evidence in mice suggest that GDNF expression levels decrease with ageing, while it might increase with stem cell depletion (65). After a treatment with low dose busulfan, GDNF expression was found to be increased and that might be necessary to restore the pool of SSCs and their subsequent proliferation (65).

However, when this factor is lacking, the germline population gradually declines, reducing its replication and increasing downstream differentiation, down to a condition where the tubules are populated solely by Sertoli cells (SCO) (61). Providing GDNF stimulation again may allow for a new expansion of the cell pool (62).

One of the potential ways in which chemotherapeutic agents might reduce GDNF expression is through DNA methylation, the main epigenetic mechanism capable of affecting male fertility. Several agents, including cisplatin (66) and doxurubicin (67) have been proven to induce important epigenetic modifications to cellular DNA. Methylation of some sequences called CpG islands, rich in dinucleotides composed of Cytosine and Guanine, is able to block access to transcription factors and reduce the expression of some genes (68, 69). This would make the employ of some demethylating agents promising, such as eicosapentaenoic acid (EPA) which is able to activate several enzymes that can counteract cytosine methylation, promoting the re-expression of silenced genes (69).

Our group has studied the effect of cisplatin, doxurubicin, and 4-Hydroperoxycyclophosphamide, chemotherapeutics known to be gonadotoxic, *in vitro* on pre-pubescent porcine Sertoli cells (70), a model known to be adequate for toxicity study (71). Drug exposure resulted in reduced expression of the GDNF gene and protein, as well as reduced expression of AMH and Inhibin B, which are markers of function in pre-pubertal Sertoli cells. In cultures treated with high dose of cisplatin and EPA, there was a recovery of GDNF, AMH and Inhibin B expression, showing a protective effect on the male gonad. Treatment with cisplatin and d 5-aza-2’-deoxy- cytidine, a known demethylating agent used in several chemotherapy protocols, allowed to obtain similar results, supporting the hypothesis of the ability of EPA to protect against epigenetic alterations of DNA and opening the future to further studies to evaluate the effect of this substance on the human pre-pubescent testis.

Despite promising evidence in animal models, guidelines do not currently include the use of protective substances during cancer therapy in children (5, 6).

## Surgical Approaches to Preserve Testicular Function

Pediatric testicular tumors are rare nosological entities, the most common being germ cell tumors, but they can occur bilaterally and synchronous or metachronous in up to 5% of cases (72). In such cases, a treatment with enucleation of the neoplasm (73, 74) instead of a total bilateral orchidectomy can be considered, if the tumor is of a small size and it is confined to the testis. Careful follow-up is necessary, as there is a risk of recurrence after enucleation of about 5%. The same approach is possible with Leydig cell tumors, in which the risk of recurrence after conservative treatment appears low (75).

There are several clinical cases reported in the literature, the first dating back to more than 30 years (76), in which a testicular transposition was performed to protect the residual gonad from adjuvant radiotherapy treatment. The healthy testis was transposed at the inguinal (77), abdominal (78) or leg region (79) and then repositioned in the scrotum at the end of therapy. It is interesting to note that in one clinical case report (80), the testis was able to resume spermatogenesis during the following months after post-traumatic repositioning in a subcutaneous pocket at tight level.

In cases of scrotal neoplasia in which extensive excision of skin and muscle layers is necessary, displacement of the testis in the contralateral hemiscrotum has been attempted to preserve its function (81). However, such approaches should be considered experimental and are not currently recommended in guidelines until further investigation (5, 6).

## Cryopreservation: Testicular Tissue Or Cell Suspension

Young peripubertal patients might be able to produce spermatozoa and a semen sample can be obtained as early as 12 years old (82). Once spermatogenesis is initiated, seminal parameters are comparable to those of adult patients (83, 84). In younger patients, in whom the sperm production has not started yet or who for whatever reason are unable to produce seminal fluid, only experimental approaches are available, such as preservation of testicular tissue obtained by biopsy or orchiectomy, when required for the treatment course of the clinical condition.

The experience of several centers both in Europe (85) and in the USA (86) is remarkable with respect to the possibility of cryopreserving pre-pubescent testicular tissue for use in approaches aimed at restoring fertility in the future. Proposed freezing protocols are numerous (86–92), including fast or slow freezing and the use of various cryoprotectants. Most centers employ slow freezing combined with the use of Dimethylsulfoxide (DMSO) to protect cells from damage (88, 93) while other facilities use DMSO and sucrose, DMSO and human serum albumin or DMSO & ethylene glycol (93). Some studies have alternatively tested vitrification, a protocol of ultrarapid freezing associated with different concentrations of cryo-protective substances, with the aim of preventing the formation of ice crystals (94).

This approach appears promising, but further studies are needed to verify its actual superiority. It is also possible to choose to freeze a testicular cell suspension, which would reduce some complications due to the freezing of a macroscopic tissue sample, such as creating an uneven cellular cooling rate. This procedure would not allow to preserve the spermatogenetic niche in its entirety (95, 96) and has also been studied in humans less intensively (97). Freezing cells rather than tissue fragments will make it impossible to employ some techniques, such as testicular graft transplantation or tissue culture, whereas a cryopreserved tissue fragment could undergo further enzymatic digestion to obtain SSCs and other testicular cells (98). It is essential to improve the freezing protocol, trying to reduce the generation of alterations in thawed sperm quality (99).

A questionnaire proposed to 24 facilities by the European Society for Human Reproduction and Embryology (ESHRE) in 2012 reported that several centers in Europe offered this possibility and have already involved 260 young patients (85). A subsequent survey in 2019 (86) brought the number of patients involved up to 1033, more than a 4-fold increase. Numerous hospital facilities in the US (86) are currently able to cryopreserve testicular tissue, and the current goal is to create networks with a well-defined common protocol to offer this possibility to as many patients as possible. It is also important to note that approximately one third of the patients enrolled had already started cycles of gonadotoxic chemotherapy, which can potentially compromise the quality of the preserved tissue.

Current guidelines (5, 6) recommend that cryopreservation of testicular tissue be performed only during approved clinical trials or experimental protocols.

## Fertility Restoration Employing Testicular Tissue

### Testicular Tissue Transplantation

One of the potential options to restore fertility in a patient undergoing gonadotoxic therapies is the transplantation of previously frozen testicular tissue. This option has been under investigation for several years and numerous studies have been published on animals, the majority of which have tested testicular tissue xenograft into adult immunodeficient nude mice. Xenotransplantation of pre-pubescent human testicular tissue into laboratory animals is not a technique that is expected to be employed to restore fertility in patients undergoing gonadotoxic therapies, due to the high risk of zoonosis transmission (100), nevertheless it is useful to study the mechanisms of transplantation and the survival of spermatogonial cells after it has been performed. Moreover, this technique could be in the future exploited to exclude the presence of neoplastic cells contamination in the testicular tissue, in preparation for a future autograft in the patient (101)

Data is available regarding xenotransplantation of tissue obtained from goats, pigs, mice (102), horses (103), cats (104), cattle (105), rhesus monkeys (106), dogs (107), hamsters (108), and rabbits (109). In all these species, once the transplanted tissue was recovered from mice and analyzed, complete donor spermatogenesis was demonstrated, and in some of these experiments (109–113) live and healthy progeny has been obtained.

Despite the undoubtedly promising results, several questions remain to be clarified. Studies that have performed xenografts of pre-pubertal human testicular tissue (92, 100, 114) have not shown appearance of complete spermatogenesis yet. There could be several obstacles, including placement of the transplant in an ectopic or orthotopic location. Early attempts at xenotransplantation, both from human and animal donors, were almost all placed in the ectopic site, but transplantation placed in the testicular site has been shown to have a higher probability of survival and maturation, probably on account of the different local temperature (114, 115). In contrast, whether the tissue is fresh or thawed from previous cryopreservation does not seem to make a difference (116, 117).

Xenograft experiments from pre-pubescent human donors have, however, demonstrated prolonged (up to 9 months) survival of SSCs and Sertoli cells, and obtained secondary spermatocytes (114) or spermatid- like cells (92).

Nevertheless, the survival of spermatogonial cells in transplants is not high (117) and it seems to be closely related to the future of tissue vascularization, which must proceed with capillary formation that is supplied by host vessels (118), since the graft is transplanted without any vascular anastomoses. To improve tissue survival, several approaches with pro-angiogenic,anti-apoptotic and anti-oxidant molecules have been attempted. The use of recombinant FSH (119) and Testosterone (120) has not shown encouraging results on testicular graft survival.

Bovine testicular tissue treated with vascular endothelial growth factor (VEGF) at the time of implantation in mice (121) was heavier at recovery than the untreated control and showed a higher percentage of seminiferous tubules containing differentiated cells. Since the early approaches, numerous experiments have begun to treat the tissue with VEGF, whether in the context of autografts in mice (122), bovine tissue xenografts (123), and even pre-pubescent human testis xenografts (124).

On the pre-pubescent human testis, *in vitro* pretreatment with VEGF appears to increase vascularization and survival of SSCs and seminiferous tubule integrity (124). Subsequent experiments (122) on autograft in mice tested the combined effect of VEGF and platelet-derived growth (PDGF) nanoparticles, showing that the combination of the two factors appears to further improve vascularization. The use of necrosis inhibitor substances also seems promising (125).

Autotransplantation, the method more desirably applicable to pre-pubertal patients undergoing gonadotropic therapies, has been tested on nonhuman primates (111, 126, 127). These studies demonstrated on marmosets (126) better survival of transplants at the orthopedic site, which achieved complete spermatogenesis, probably because of reduced scrotal temperature compared with other body regions, and better results of tissues taken from pre-pubertal animals compared with adults, perhaps because of greater resistance to hypoxia. A study in rhesus monkeys (127) showed the achievement of complete spermatogenesis after orthotopic autotransplantation of testicular tissue, subjected to cryopreservation for a period longer than two years. A subsequent study in rhesus monkeys (111) showed complete spermatogenesis obtained from autologous transplants of testicular tissue placed subcutaneously either in the scrotum or behind the back, cryopreserved or fresh. Spermatozoa were also shown to fertilize oocytes, and *via* ICSI viable and healthy offspring were generated.

In a study of autotransplantation of testicular tissue in mice (128), alginate-encapsulated tissue with or without the addition of VEGF nanoparticles also appeared to improve spermatogonial recovery post-transplantation.

It should be emphasized that testicular tissue transplantation is not able to restore fertility in the recipient in the absence of medically assisted procreation, since it has not been proven that the graft is able to create anastomoses with the seminal tract, thus leading to ejaculation of spermatozoa with seminal fluid and fertilization during natural sexual intercourse. As today, these methods appear to be entirely experimental and have not yet been tested on human patients, either pre-pubescent or adult.

### Spermatogenesis *In Vitro* From Testicular Tissue

Achieving spermatogenesis *in vitro* from testicular tissue would allow to avoid the risks related to other methods, in particular the transmission of zoonosis *via* xenografts (100) and the possible neoplastic contamination of tissues obtained from cancer patients (101, 129–132). This approach has been studied for many years (133, 134), but it is still experimental, and the current goal is to optimize culture systems in order to further progress in this direction (135).

In mouse, *in vitro* spermatogenesis has been obtained from testicular tissue cultures (135–137) and these spermatozoa were found to be able of fertilizing embryos and producing healthy offspring. Sato et al’s (137) experiments developed a culture system called “*in vitro* transplantation” (IVT), in which SSCs from one donor are injected into the empty seminiferous tubules of another animal, and the result is incubated in a culture system. In other studies (138) the air- liquid interphase method has been used, obtaining competent spermatozoa capable of generating healthy and fertile offspring, even exploiting previously cryopreserved tissue. The quality of spermatozoa obtained with such cultures has been evaluated (139), showing that the majority of them are characterized by normal haplody, non-fragmented DNA and condensed chromatin.

Full *in vitro* spermatogenesis was reached even in bovine (140) and rat (141) testicular tissue culture

Obtaining human spermatozoa *in vitro* has proven more challenging. Numerous attempts have been made to understand the best culture conditions of testicular fragments (142), investigating proper temperature, serum, and wheter gonadotropin stimulation is necessary.

So far, postmeiotic haploid cells have been obtained from pre-pubescent human testicular tissue fragments, both in organotypic culture (143) and exploiting a 3D culture system (144). One study (145) obtained haploid spermatids from SSCs obtained from testes of cryptorchid patients cultured in 2D systems enriched with arachidonic acid and stem cell factor (SCF). Such spermatids were able to fertilize murine oocytes by Microinjection of round spermatids (ROSI).

Recreating the complex microenvironment of the spermatogenic niche seems to be essential to achieve progress (135, 146) so there has been a clear shift towards 3D culture systems over the old 2D systems. Also, the potential of the culture to generate an intact and functioning blood-testicular barrier (147) seems to be relevant, as occurs *in vivo* during puberty.

## Fertility Restoration Employing Cell Suspension

### SSCS Transplantation

Different approaches exploiting testicular cell suspension are under study. A promising one is SSCs autotransplantation. This mechanism has been described since 1994 in mice (148) and over the years has been the subject of numerous studies on different experimental animals, also it appears to be the only one potentially able to restore fertility without the need to employ medically assisted procreation. The ability to colonize seminiferous tubules, as well as the possibility to initiate spermatogenesis, is related to the amount of SSCs transplanted (149). Furthermore, it has been estimated that only 10% of transplanted spermatogonial stem cells are able to form colonies (149). Such cells are rare, representing approximately 1 in 3500 cells in the adult mouse testis (150), and the amount of testicular tissue that can be harvested in the pre-pubescent would not be sufficient to provide an adequate number of cells.

For this reason, several methods have been developed to generate efficient culture systems of SSCs, amplifying their number *in vitro* before transplantation, and this approach has been initially studied in mice (151). The collected testicular tissue undergoes enzymatic digestion in several steps according to well- defined protocols (152, 153) and great attention has been paid to find a method that allows to efficiently isolate SCCs as soon as this stage (154).

A further complication is the difficulty in identifying SSCs, based on the markers they express and the proteins they produce (155, 156) since a large proportion of them are also expressed by testicular somatic cells and differentiating them appears complex (157). The ability to characterize these cells, purify and amplify them is essential for successful colonization of the seminiferous tubules in the recipient. Stage- specific embryonic antigen-4 (SSEA-4) (158) is one of the many promising markers of this cell population. However, in recent studies, this marker has shown reduced expression in quiescent State 0 cells, making SSEA-4 less suitable for the isolation of SSCs. The search for the most appropriate marker remains ongoing (159).

Many potential growth factors to achieve adequate proliferation of these cells have been extensively evaluated (160–163), including proposed leukemia inhibitory factor (LIF), epidermal growth factor (EGF), basic fibroblast growth factor (bFGF), Insulin like grow factor 1 (IGF-1), Colony stimulating factor 1 (CSF-1) and the importance of GDNF, and the possible addition of its soluble receptor alpha-1 in culture has been demonstrated (160). The required growth factors appear to be identical in rats and mice (164) and therefore some kind of conservation between species has been hypothesized.

*In vitro* proliferation of SSCs obtained from different animals, including mice and rats (151, 164, 165) porcine (166), cattle (167) and tree shrew (168), has been achieved. The same approach has allowed *in vitro* proliferation of human SSCs, taken from testicular tissue obtained from pre-pubertal patients during orchidopexy (169), for cryopreservation in cancer patients (170) or from adult patients undergoing orchiectomy (171), from patients with obstructive or non-obstructive azoospermia (172) or from deceased organ donors (158).

Using subcultures, human adult SSCs were cultured and propagated up to 28 weeks and their numbers increased more than 18,000-fold (171). The proliferation capacity of SSCs from pre-pubertal patients seems to be even higher (169). Nevertheless, the long-term fate of SSCs cultures seems unclear. Several promising studies have been carried out to elucidate the best culture conditions, but many of them have not characterized SSCs with suitable surface markers, nor defined the ideal conditions for promoting the development of cells at different stages of maturation. More recent work has been able to identify the full gene expression of SSCs and to assess the molecular pathways activated in their proliferation. This approach appears useful for better understanding their development and improve our culture system (173).

Cancer patients, especially those with hematological diseases, may harbor neoplastic infiltrates in the testicle, as shown in pre-treatment biopsies of children with Acute Lymphocytic Leukemia (174). Such neoplastic cells if transplanted can give rise to new neoplasms (101, 129–132) so it is essential to ensure purification of the SSCs sample. It has been proven that in rats it is enough to transplant in the testis only 20 leukemic cells, mixed with germ cells, to initiate a relapse of the disease (175).

The most studied mechanics so far are culture systems (176), Fluorescence-activated cell sorting (FACS) (129, 130, 132), and Magnetic-activated cell sorting (MACS) (131, 177) but their evaluation has shown conflicting and sometimes not sufficient results to ensure the safety of the method, making further studies necessary.

The first approach for SSCs transplantation was characterized by multiple microinjections into the seminiferous tubules of the recipient mouse (148), a procedure that required open surgery with exteriorization of the testis and reflection of the vaginal tunica. Afterwards, different approaches were tried on dissected mouse, bovine, monkey and human testes (178), attempting injection of SSCs into the efferent duct or into rete testis network under ultrasound guidance, the latter method being the most promising. Some studies on human testis obtained from cadavers have tested injections of contrast agent (179) or murine SSCs (180, 181) to study the best possible operating conditions, showing that a single injection into rete testis network seems to be effective (179) and that it is necessary to find the right filling pressure, perhaps using an infusion pump, to adequately fill the tubules and reduce fluid leaking into the testicular interstitium (180, 181).

Allografting of SSCs has been tested in sheep (182), goats (183), and nonhuman primates (47, 184), generating healthy live offspring. Only one human clinical trial is reported (185), in which some adult patients who had cryopreserved SSCs prior to chemo-radiotherapy treatment underwent transplantation of such cells with intra-testicular injections. Unfortunately, there are no reports on subsequent follow-up and their seminal parameters (186).

The main doubts to be dispelled, concern the safety of these protocols and the absence of major alterations in the progeny. In mouse SSCs allografts, first and second-generation offspring appear to develop with comparable weight and height to controls and do not appear to show differences in methylation patterns of maternal, paternal, or non-imprinted genes (187). However, seminal parameters after transplantation were worse than controls, with reduced sperm concentration and motility (188). A subsequent study on a similar murine allograft showed no notable genetic alterations in either spermatozoa or progeny (189), such as chromosome number alterations, deletions, or amplifications.

### *in vitro* Spermatogenesis From SSCS and From *De Novo* Testicular Morphogenesis

Cryopreserved or fresh SSCs suspension could also be used to try to achieve *in vitro*-spermatogenesis, in specific culture systems. A type of approach is developing culture systems in which injecting SSCs, exploiting different types of matrixes, such as soft agar or methylcellulose (190) or microfuidic system (191) that allowed to obtain functioning spermatozoa in some studies.

The construction of testicular organoids (192) seems promising to create the right supportive environment for the development of SSCs. A wide variety of proposals is available, including models relying on extracellular matrix (ECM) (193) or ECM- free (194), the use of microwells (195) or 3D printing with particular bio-inks (196). A scaffold-based and scaffold-free approach has also been applied to generate human testis organoids (197) and this strategy opens the way to new future prospectives.

A different method that has been studied, is performing, under the back skin of immunodeficient mice, a graft of testicular cell suspension containing other cells besides SSCs, including Sertoli cells, Leydig cells and peritubular myoid cells, in a definite proportion (198). Such a cell mix seems to be able to organize into a testis-like structure, *via* a complex process that has been named *de novo* testicular morphogenesis, generating a spermatogenic niche and recovering steroidogenic capacity, up to complete spermatogenesis (198). This approach has been studied utilizing cells obtained from rodents (199, 200) zebrafish (201), sheep (202) and cattle (203), as well as from pigs (198, 204). Some of these studies have included the cell suspension in matrices as scaffolds to support their growth (205, 206). Seminiferous tubule formation has also been noted after cell suspension grafting inside the testis of rhesus monkeys (207) with resumption of donor spermatogenesis.

This possibility seems to be very interesting to study the interactions between different testicular cell types and to better understand the mechanisms of gonadal development (203, 205).

## Conclusion: Future Challenges and Promising Methods

During the last years we have witnessed a swift progress in studies regarding potential approaches to preserve and restore male fertility, but few of these methods are currently clinically applicable in the prepubertal oncological patient. Current clinical guidelines and approaches involve prompt counseling with a reproductive medicine specialist, reduction of gonadotoxicity of the chosen therapy when possible, and potential participation in experimental protocols where offered.

Cryopreservation of testicular tissue or cell suspension is offered in the context of experimental protocols in several centers around the world, which have developed shared methods and a considerable experience on this field, however there is still no certainty about which are the best methods and the potential damage to sperm quality. Cryopreserved testicular material, either tissue fragment or cell suspension, has shown in several experimental animals the ability to re-initiate spermatogenesis and even to generate healthy living offspring, but there is not yet sufficient evidence in humans. Out-of-body approaches, such as *in vitro* spermatogenesis, are promising but early in their development. We believe that there is a need to pursue these approaches, while continuing to evaluate the potential efficacy of numerous chemicals and pharmacological substances that could help to protect the delicate prepubescent testis from the insult of oncological therapies.

## Author Contributions

All authors listed have made a substantial, direct, and intellectual contribution to the work and approved it for publication.

## Funding

This research was founded by Fondazione CARIT Cassa di Risparmio di Terni, code or Project FCTR21UNIPG. EE is a recipient of an University of Perugia PhD research grant

## Conflict of Interest

The authors declare that the research was conducted in the absence of any commercial or financial relationships that could be construed as a potential conflict of interest.

## Publisher’s Note

All claims expressed in this article are solely those of the authors and do not necessarily represent those of their affiliated organizations, or those of the publisher, the editors and the reviewers. Any product that may be evaluated in this article, or claim that may be made by its manufacturer, is not guaranteed or endorsed by the publisher.
